# Lymphatic filariasis in Peninsular Malaysia: a cross-sectional survey of the knowledge, attitudes, and practices of residents

**DOI:** 10.1186/s13071-014-0545-z

**Published:** 2014-11-27

**Authors:** Nazeh M Al-Abd, Zurainee Mohamed Nor, Abdulhamid Ahmed, Abdulelah H Al-Adhroey, Marzida Mansor, Mustafa Kassim

**Affiliations:** Department of Parasitology, Faculty of Medicine, University of Malaya, Kuala Lumpur, 50603 Malaysia; Department of Biology, Faculty of Natural and Applied Sciences, Umaru Musa Yar’adua University, Katsina, Katsina State Nigeria; Department of Anesthesiology, Faculty of Medicine, University of Malaya, Kuala Lumpur, 50603 Malaysia

**Keywords:** Lymphatic filariasis, Mass drug administration, Control program, Mosquito

## Abstract

**Background:**

Lymphatic filariasis (LF) is a major cause of permanent disability in many tropical and sub-tropical countries of the world. Malaysia is one of the countries in which LF is an endemic disease. Five rounds of the mass drug administration (MDA) program have been conducted in Malaysia as part of the Global Program to Eliminate Lymphatic Filariasis (GPELF) by year 2020. This study investigated the level of awareness of LF and the MDA program in a population living in an endemic area of the country.

**Methods:**

A descriptive cross-sectional survey that involved 230 respondents (≥15 years old) living in the LF endemic communities of Terengganu state in Peninsular Malaysia was performed. Demographic, socioeconomic, and knowledge, attitudes and practices (KAP) data of the respondents were obtained using pre-tested questionnaires and were analyzed using SPSS software version 13.0.

**Results:**

More than 80% of the respondents were aware of LF and the common symptoms of the disease. Moreover, about 70% of the respondents that were aware of LF indicated that it is a problematic disease. Approximately 77% of the respondents indicated that filariasis is transmitted by mosquitoes. Two-thirds of respondents preferred hospital treatment for illness; however, only 12% had participated and/or received treatment for LF during an MDA program. Only 35% of the respondents that participated in this research were aware of the MDA program that had taken place in the area. None of the respondents had knowledge of the drug used in the treatment of LF. The findings from this research indicated that there was no significant association between LF awareness and with gender, age group, educational status, occupation, or socio-economic status of the respondents (*P* >0.05).

**Conclusion:**

A good proportion of the respondents are aware of LF, its mode of transmission and symptoms, however they demonstrated a poor knowledge of MDA which took place in the study area. For greater understanding of LF in the Malaysian population, there is a need for an enhancement in the delivery of health education and information programs and mass mobilization campaigns in endemic communities.

## Background

Lymphatic filariasis (LF), often called elephantiasis, is considered by the WHO as the second most common debilitating mosquito-transmitted disease caused by filarial parasites [1]. It is classified as one of the neglected tropical diseases (NTDs) by the World Health Organization (WHO) and is the second leading cause of permanent long-term disability in the world [1,2]. The prevalence of LF continues to increase, and LF is a major public health concern that is associated with significant socio-economic obstacles [3]. Recent estimates suggest that approximately 1.4 billion people living in 73 tropical and sub-tropical countries are at risk of infection [4]. It has been estimated that approximately 120 million people have been infected globally by the disease and that approximately 40 million have become incapacitated due to the disease [4,5]. Approximately 65% of those infected live in South-East Asia, 30% in Africa, and the remainder in other tropical areas [6]. Approximately 90% of LF infections are caused by *Wuchereria bancrofti* and the rest are caused by *Brugia malayi* and *B. timori* [4]. In these endemic regions, the psychological, economic, and social impact associated with the disease is significant, adversely affecting productivity and quality of life. The most common manifestations of LF are hydrocele, lymphedema, and elephantiasis. In 1997, the WHO organized the Global Programme with the aim of eliminating LF as a public health crisis by the year 2020, mainly through the institution of annual mass drug administration (MDA) programs for those people living in endemic areas [7,8].

In Malaysia, LF is caused by *W. bancrofti* and *B. malay*i and is transmitted by mosquitoes of the genus *Anopheles* and *Mansonia* [9]. It occurs only in very small pockets in Malaysia: Sabah, Sarawak, and several states of the Peninsular Malaysia including Terengganu, Kelantan, Pahang, Selangor, and Johor [9]. In Malaysia, two phases of transmission-assessment survey (TAS) were performed during 2010–2011, with the goal of eliminating LF by 2015 [10]. According to the Ministry of Health Malaysia, five rounds of MDA program have been completed in all endemic areas between 2004 and 2008, with >80% coverage, using diethylcarbamazine (DEC) and albendazole [10]. According to Dr. Rose Faiza Hanim, the manager of the LF control program in Malaysia, the MDA program was strictly conducted according to WHO guidelines. TAS survey in Malaysia was conducted mainly in the Sabah state. After TAS-1, it was observed that the number of positive cases still exceed the critical cut-off value. Hence, MDA was continued before re-testing in TAS-2, but only one round of the drug administration was conducted due to DEC supply problems. TAS-2 was thus conducted after administering the Brugia rapid test (BmR1) and the result still showed values higher than the critical cut-off values and therefore it was recommended that MDA should continue in Malaysia (8). Despite these efforts, reports indicate increasing incidence of the disease. Thus, knowledge, attitudes, and practices (KAP) studied and additional TAS and MDA programs are required in the study area and other LF endemic areas of the country.

The success of the MDA program is dependent on the knowledge of the intended recipients of the program and is dependent on the program delivery system. Knowledge plays an important role in the prevention of LF. Awareness of LF is a suitable method to avoid the disease and remain healthy, as it is known that misunderstanding of illness and health-seeking behavior may improve or interfere with the effectiveness of control measures [11]. Therefore, we conducted a study of the population living in an LF endemic area in Terengganu state, Malaysia. The aim of the study was to assess the knowledge, attitudes, and practices of the study population with respect to LF, as well as knowledge of the MDA program. The results of this survey will aid in the design and implementation of educational strategies, as well as in the development of disease control and interventional methodologies that require active community participation.

## Methods

### Description of the study area

**Figure 1:**
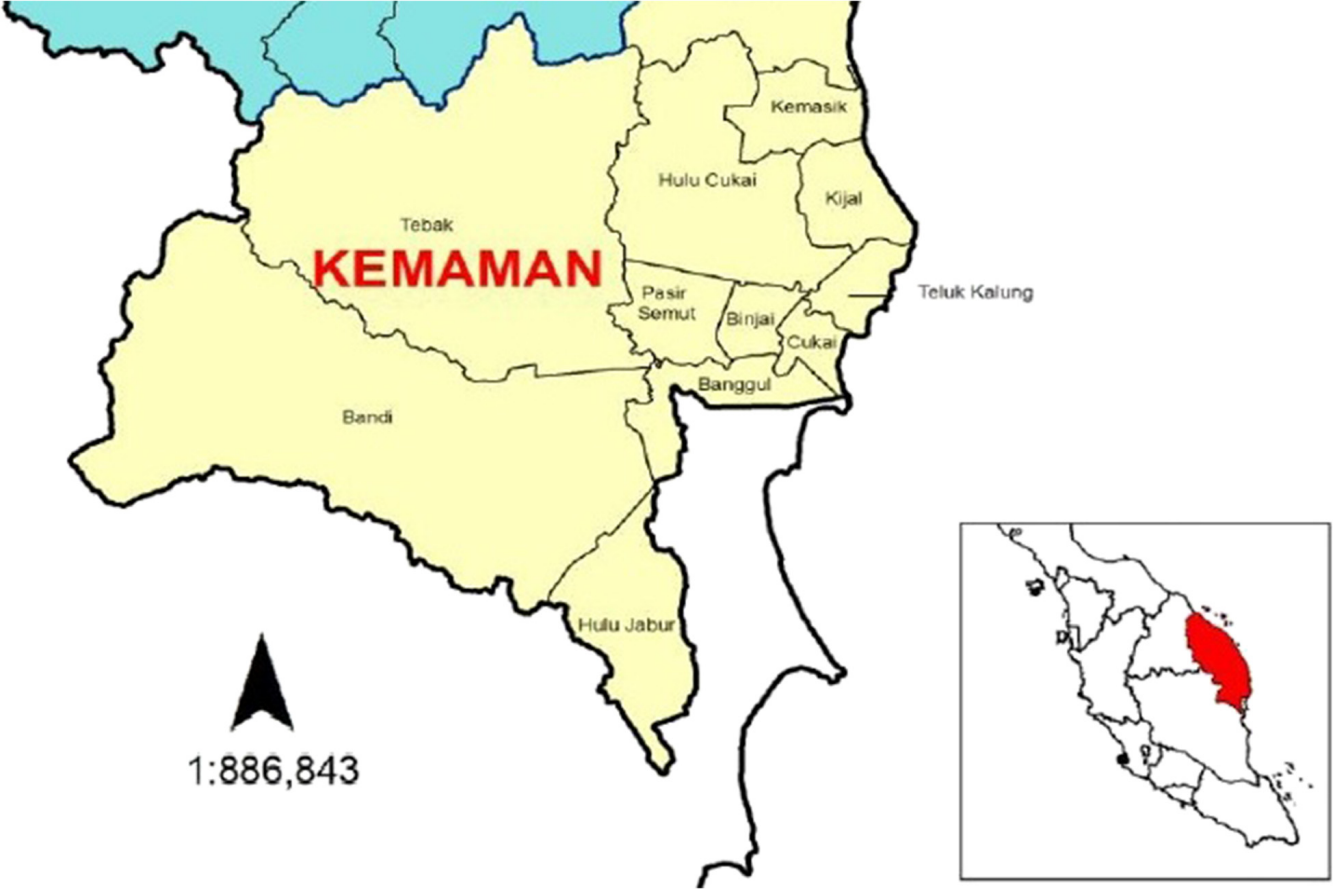
**Location of the study area.** The inset panel shows the area of Peninsular Malaysia in which the Kemaman district is found.

This cross-sectional study was conducted in Kemaman district, which is located in a coastal area of Terengganu state in Malaysia (Figure 1). The area was selected on the basis of an established occurrence of LF within the region. According to the Ministry of Health [10], the microfilariae (mf) rate in the endemic areas of the country ranges from 1.41 to 2.14 to per 1000 people, with 387 cases reported in 2011. The capital of the district is Cukai town, which is a coastal town located at latitude 4°14′N and longitude 103°25′E and is at an elevation of 42 feet above sea level. According to the 2006 population census of Malaysia, the Kemaman district had a population of 174,876 people, with Cukai town having a population of 82,425 people. Other settlements in the district include Hulu Cukai, Kijal, Seri Bandi, and Ibok. The main occupations of the residents include fishing, subsistence farming, transportation, industrial labor, and public service.

### Study population

The study was conducted between August and October 2012. The study population consisted of all adolescents and adults who attended the participating health clinics within the study area during the survey period and agreed to participate in the survey. The clinics were the Sri Bandi health clinic, the Ibok health clinic, and the Kijal health clinic, which are the main public health facilities in the study area. In order to participate in the study, respondents had to have lived in the area for at least 5 years and had to be 15 years or older. In addition, pregnant women were excluded from the study to avoid partial or non-compliance. A total of 230 volunteered respondents aged ≥15 years participated fully in the survey. The survey was conducted with the support and cooperation from the local community leaders and medical personnel in the area.

### Study questionnaire

A semi-structured questionnaire was developed by the researchers with input from a medical anthropologist. The questionnaire was validated and pre-tested with 20 individuals to ensure reliability and validity prior to initiating the fieldwork. During the fieldwork, questionnaires were administered with the help of a medical doctor and a nurse who were indigenous to the research area. The study participants were interviewed, using the local language, to determine the extent of each participant’s knowledge of LF, including prevention, treatment, symptoms, and transmission, as well as the attitude of the participants towards the disease. Additional questions included those about the MDA program, such as participation in the MDA program and the source of information about the MDA program. Some questions were open ended and allowed the respondents the chance to give greater details while others were restricted to a yes or no answer. The questionnaire was not distributed house-to-house. The questionnaire used in the survey was written in Bahasa Melayu, the national language of Malaysia.

### Ethical consideration

All respondents were fully notified that participation was voluntary and that it was possible to withdraw from the research without notice. Those who wished to participate were required to sign a consent form prepared in accordance with the guidelines of the Malaysian Department of Health and Human Services prior to the administration of the questionnaires.

### Statistical analysis

The data obtained for this research was analyzed using SPSS (Statistical Package for Social Sciences, Version 13.0; SPSS, Chicago, IL, USA; 2004). The data was cleaned and checked thoroughly to ensure correctness of entries before the initiation of analysis. The demographic and socio-economic characteristics of the respondents were presented in percentages and frequencies. Association of the knowledge of filariasis with demographic factors of the respondents was assessed using the chi-square test. A *P*-value of less than 0.05 was considered to be significant in the determination of association between the variables.

## Results

### Study population characteristics

A total of 230 people voluntarily participated in the survey; 68.7% of respondents were female and 31.3% of respondents were male. The general socio-demographic characteristics of the respondents were presented in Table 1. Most patients were aged 30–39 years, while the age group ≥60 years had the least number of patients. Of the respondents, 90% had received education: 37% at the primary level, 39.1% at the secondary level, and 11.3% at the tertiary level. Approximately 12% of the respondents had not received any formal education. Almost half of the respondents (47%) were employed, with 70% of the employed respondents earning more than RM500 (about US$165) per month. Of those not employed, the majority were housewives. There was an equal split in the percentage of respondents who owned wood/bamboo-based and the percentage of respondents who owned brick-based houses.

**Table 1:** **Socio-demographic characteristics of the study population**

**Variable**	**Number (N = 230)**	**Percent (%)**
**Sex**		
Male	72	31.3
Female	158	68.7
**Age**		
15–29 years	67	29.1
30–39 years	90	39.1
40–49 years	58	25.2
50–59 years	10	4.3
≥60 years	5	2.2
**Educational level**		
No formal education	28	12.2
Primary	86	37.4
Secondary	90	39.1
Tertiary	26	11.3
**Occupation**		
Employed	108	47.0
Unemployed or housewife	122	53.0
**Type of house**		
Bamboo/wood	131	57.0
Bricks/rock	99	43.0
**Income level**		
≤ RM 500.00 ($160.00)	61	26.5
> RM 500.00 ($160.00)	169	73.5

### Knowledge of lymphatic filariasis

Almost all of the respondents (83.9%) had heard about LF and reported that the source of LF information was school (30.1%), mass media (21.8%), or both (8.8%). Others had heard about LF from health centers (12.9%), mass media and health centers (11.4%), or from other people (15.0%). The majority (77.2%) of respondents knew that LF is transmitted by mosquitoes. Approximately 20% did not know how LF is transmitted and 1 respondent mentioned bacteria as the agent of transmission. Slightly more than half (59.6%) reported that the main symptom of LF was swollen legs, while 11% admitted not knowing any symptoms. The knowledge of the respondents regarding LF and its transmission is shown in Table 2.

**Table 2:** **Respondents’ knowledge of lymphatic filariasis, its transmission and MDA**

**Variable**	**Number (N = 230)**	**Percent (%)**
**Knowledge about lymphatic filariasis**		
Yes	193	83.9
No	37	16.1
**Source of knowledge**		
Mass media	42	21.8
School	58	30.1
Health center	25	12.9
Mass media and school	17	8.8
Mass media and health center	22	11.4
Other people	29	15.0
**Assumed method of transmission**		
Bacteria	1	0.5
Mosquito	149	77.2
Worms	4	2.1
Don’t know	39	20.2
**Knowledge of mass drug administration**		
Yes	81	35.2
No	149	64.8
**Source of knowledge**		
Mass media	26	32.1
School	32	39.5
Health centre	18	22.2
Mass media and school	1	1.2
Mass media and health centre	4	5.0

### Attitudes and practices towards LF and its treatment

Approximately 68% of the respondents that exhibited LF knowledge in the survey perceived LF to be a problem. Furthermore, approximately 41.5% of the respondents perceived LF to be a medical problem that results in symptoms including pain, fever, itching, and the inability to walk. In contrast, approximately 40% of respondents viewed LF to be an economic problem that occurs as a result of inability to work, loss of employment, and expenditures for medication and/or transport to health facility. Approximately 11.5% of respondents viewed LF to be a social problem, as those infected are unable to interact with the community. The remaining 8% of survey respondents did not specify the kind of problem they perceived LF to be.

When asked about treatment of illness, the majority of respondents (60.9%) preferred hospital treatment. Approximately 2% preferred to use “bomoh/dukun” (traditional healer) to treat illness. The remaining respondents (37%) preferred to combine the 2 forms of treatments. Almost all respondents had poor knowledge of the drug used in the treatment of LF, as 96.1% of the respondents indicated having no knowledge of the drug used for treatment of LF and 4% of respondents mentioned paracetamol as the possible drug used for LF treatment. Moreover, when the respondents were asked about participation in an MDA program, only 12% admitted having participated in an MDA program previously or had family member(s) treated for LF through an MDA program.

To prevent transmission of LF, more than 40% of respondents reported using protective clothes and sleeping under bed nets to protect themselves from mosquito bites. However, 15% did not indicate specific protection against mosquitoes. Most respondents were aware that cleaning of water containers, provision of good drainage, the use of chemical sprays, or a combination of these activities were the correct methods to control the mosquito population. The attitudes of the respondents towards mosquitoes are presented in Table 3.

**Table 3:** **Respondents’ understanding of the symptoms of LF, treatment seeking behavior and attitudes towards mosquitoes**

**Variable**	**Number**	**Percent (%)**
**Known symptoms of LF (N = 193)**		
Fever	8	4.1
Swollen legs	115	59.6
Body pain	1	0.5
Gland enlargement	3	1.6
Fever and swelling	29	15.0
**Do you consider LF to be a problematic disease? (N = 193)**		
Yes	130	67.4
No	5	2.6
Don’t know	58	30.0
**Type of problem (N = 130)**		
Medical	54	41.5
Economical	52	40
Social	15	11.5
Unspecified	9	7
**Preferred treatment method (N = 230)**		
Hospital	140	60.9
Bomoh/dukan/traditional healers	5	2.1
Hospital and traditional healer	85	37.0
**Presumed drug in the treatment of lymphatic filariasis**		
Paracetamol	9	3.9
Don’t know	221	96.1
**Participation of respondent or family member(s) in mass drug administration program**		
Yes	27	11.7
No	203	88.3
**Protection from mosquito bites**		
Wear clothes	25	10.9
Use of bed nets	77	33.5
Wear clothes and use of bed nets	90	39.1
No response	38	16.5
**Control of mosquitoes**		
Cleaning of water containers	30	13.0
Good water drainage	12	5.2
Use of chemical spray	15	6.5
Cleaning and drainage	42	18.3
Cleaning, drainage, and chemical spray	77	33.5
No response	54	23.5

### Knowledge of MDA program among the respondents

The majority of the respondents (65%) were not aware of the existence of the MDA program (Table 2). The remaining 35% of respondents, who had previous knowledge of the MDA program, stated that they had learnt about it from schools (40%), mass media (32%), and health centers (22%).

### Association between some demographic factors of respondents and knowledge of MDA and LF

The association between demographic factors and knowledge about LF and the MDA program among the study population was determined using chi-square tests (Table 4). No significant association was detected between the demographic factors examined and knowledge of LF or the MDA program.

**Table 4:** **Association between lymphatic filariasis knowledge and respondents’ demographic factors**

**Variables**	**Knowledge of lymphatic filariasis**		
	**Prevalence (%)**	**Odds ratio (95% confidence interval)**	***P-*** **value**
**Sex**			
Female	86.1	1.19 (0.895, 1.570)	0.130
Male	79.2	1	
**Age group**			
≥40 years	89.0	1.56 (0.818, 2.968)	0.104
<40 years	81.5	1	
**Education level**			
≥6 years formal education	85.1	1.10 (0.934, 1.294)	0.138
No formal education	75.0	1	
**Occupation**			
Employed	87.4	1.33 (0.859, 2,055)	0.114
Unemployed or housewife	80.7	1	
**Monthly income**			
≤ RM 500.00 ($160.00)	90.9	1.36 (0.188, 9.828)	0.611
> RM 500.00 ($160.00)	87.7	1	

## Discussion

This survey was conducted in the state of Terengganu which is known to be endemic for LF. The information gathered for the purpose of this survey was obtained from visitors to the clinics in the survey area. The results showed that the majority of the respondents were women which may pose potential bias. However this is not the plan of the researchers, and is not expected to affect the findings since both the women and men have equal chances of getting infected. Moreover, experienced medical personnel were used to assist in data collection especially the administration of the questionnaires. This is done because these personnel were mostly indigenes of the area, they speak the local language of the respondents and we feel that because they interact directly with the target population, involving them will facilitate compliance and cooperation of the respondents to give honest information required [10]. Furthermore, all aspects of the survey were conducted in close supervision by the researchers.

The WHO has recommended the implementation of knowledge, attitudes, and practices (KAP) surveys as a cornerstone for health promotion campaigns, as the surveys help programs adjust health education messages to increase public knowledge and awareness [12]. The KAP related to LF infection differs between regions and is heavily influenced by socio-cultural settings. Little is known about how individual communities incorporate knowledge of the origins and impacts of LF into local knowledge systems [13]. To the best of our knowledge, this is the first KAP study of LF in residents of LF endemic areas of Peninsular Malaysia.

This survey of indigenous adults who have lived in the area for at least 5 years was the first to be performed in this LF endemic area of Malaysia. The survey was limited to only those who attended the clinics for any reason; however, all pregnant women were excluded from the study. The majority of the survey respondents were female respondents, likely due to the fact that women in the area make more hospital visits or that women in the area are more cooperative and willing to volunteer for surveys. After receiving training from the researchers, medical doctors and nurses administered the questionnaires to ensure unbiased reporting and responses from the subjects.

Our study revealed that although the study area is categorized as an LF endemic area, the majority of the respondents were not aware of that status, revealing that information about the disease was not effectively conveyed to the general public. Thus, there were people who had poor or no knowledge of LF. This finding is in agreement with several previous studies performed on the population of endemic areas in Thailand [14], Ghana [15], Tanzania [16], and India [17,18].

In the control or elimination of a disease, the population involved must have prior knowledge of the disease for the control measure to be successfully implemented. Our survey, as well as others [19,20], indicated that the major sources of information were schools, health centers, and the mass media. In order to achieve greater awareness in the community, additional informational campaigns should be considered, including house-to-house visits.

In our survey, the majority of the respondents indicated knowing that LF is transmitted by mosquitoes. This is in agreement with the findings of previous studies [20,21], however, several other studies [18,22,23] have reported that the majority of respondents did not know that mosquitoes are the vectors that transmit LF. The implication of this deficit of knowledge is that families may not take appropriate measures to protect their family members, which could counteract efforts to control the disease.

In our study, the majority of the respondents recognized that the common symptoms of LF include swelling of the legs, as well as other symptoms including fever. This is consistent with previous studies [12,20,21,24,25]. In contrast, it has also been, reported that the majority of respondents in 1 study did not know the symptoms of LF [17].

Our findings with respect to the attitudes of the respondents towards LF showed that the majority of respondents view LF to be a problematic disease. Furthermore, the respondents had differing views in terms of the significance of LF as a problem, with respondents viewing LF as a medical, a social, or an economic problem. This finding is in agreement with the findings from a recent Indonesian survey [26]. The fact that only two-thirds of the respondents indicated that they view LF to be a problem shows that awareness and knowledge of the disease in general is lacking among the residents of this endemic community. Thus, there is a need to increase efforts to improve education of the residents to ensure effective control of LF.

Our survey revealed that the majority of respondents preferred hospital treatment during illness, indicating that there is awareness of the usefulness of hospitals. However, approximately 40% of the respondents still consider traditional methods when treating illnesses. Similarly, in Nigeria, the majority of respondents were reported to prefer hospital treatment, while a small portion preferred traditional treatment methods [20]. Although, most of the respondents preferred hospital treatment, their knowledge of the drug used in the treatment of LF was poor, similar to what has been observed previously in India [18].

Despite the fact that the study area is known to be endemic for LF and an MDA program was previously conducted in the area, our survey showed that only a small proportion of respondents had obtained treatment for LF. This result could suggest that the respondents are either ignorant of or are taking for granted the treatment of LF and the MDA program. It could also be possible that the drug deliverers do not strictly observe the people taking their drugs directly. As approximately two-thirds of the respondents were not aware of the MDA program that took place in the area and approximately one-fifth had not heard of LF before the survey, it is likely that a large proportion of people did not participate in the MDA program. Similarly, the poor awareness of the people regarding the MDA program results in poor participation. Thus, the success of an MDA program depends upon the target population’s knowledge of the benefits. Knowledge is therefore a vital component in the success or failure of any MDA program [11]. Poor knowledge leads to poor participation, and poor participation leads to low coverage and persistence in transmission of the disease. Moreover, we observed that the MDA program in Malaysia concentrated mainly on distributing the drugs to people, with less emphasis on ensuring that they actually swallowed the drugs or that they are educated on preventive measures such as the use of bed nets and care of enlarged limbs. Hence, suspected patients kept on going to the hospitals with one complain or the other. However, in this survey we did not encounter any admitted case of LF in any clinic.

One of the most important preventative measures in the eradication of mosquito-borne diseases, such as filariasis, is the prevention of mosquito bites. Our study indicated that the majority of respondents use protective clothes or sleep under bed nets to protect against mosquito bites; however, approximately one-sixth of the respondents did not mention using any form of protection. The fact that a proportion of respondents did not mention any protective measures probably indicates that the respondents did not see the need for protection or that they are not comfortable taking preventative measures. There could be some other barriers too such as cost, availability or ease of use of the materials. Either way, the lack of knowledge with respect to the transmission of LF is apparent. Interestingly, our survey revealed that most of the respondents were aware of several ways used to control mosquito breeding, demonstrating some understanding of vector control strategies, although this knowledge was not necessarily translated to be part of an effective eradication program. While our results are in agreement with a previous study [20,27], a number of previous studies demonstrated that the majority of their subjects did not know the importance of minimizing mosquito contact in preventing infection [13,16,23,26].

As evidenced from the findings of a previous survey, the knowledge gap regarding LF, as well as general attitudes towards and perceptions of the eradication program, was the basis of the major causes of lower compliance [28], this could have likely resulted in the continued endemicity of LF in the endemic areas of Malaysia. This has been shown to occur in Kenya [29,30], Papua New Guinea [31], and in India [25,32], where it was reported that there was low compliance for an MDA program due to poor knowledge of LF by the target population. On one hand there is sometimes the problem of poor drug delivery. This was observed to be among the hindrance encountered in Malaysia after the completion of TAS −1 when MDA was continued [8].

Furthermore, as observed by some other researchers, no single formula can ensure success of MDA in all settings as compliance may be negatively affected by other factors such as the perceptions of the potential benefits of participation, the possible risk of adverse events as well as the fear of the unknown by the target population [10].

The success of the MDA program to treat LF is dependent on the knowledge of the target population. It cannot be assumed that the distribution of information from schools, health centers, and mass media is sufficient at conveying the information effectively. Recent studies have shown that the distribution of information leaflets and posters [25] are effective. The use of appropriate means of communication based on prevailing conditions is important in ensuring that messages reach the target audience.

There was no significant association found between LF knowledge and gender, occupation, age, educational status, or income of the respondents; however, our results did indicate that females, older respondents, employed respondents, and respondents with higher income had greater knowledge of LF. This finding is in contrast to the results of a study performed in the Philippines [13] that found significant associations between gender, age, and educational status of respondents with LF knowledge.

Despite the fact that LF is an increasing burden from the perspective of both public health and economics, there seems to be little research on LF in comparison to other neglected tropical diseases. Public health authorities therefore have a great role to play in educating the people living in endemic areas for LF on the dangers posed by the disease in terms of loss of DALYs, causing permanent incapacitation to patients and huge economic loss on treatment etc., which leads to a general low productivity. The effect of LF is also very serious on the part of the governments, as considerable funds are needed for both MDA administration and case management in endemic areas. There is thus, an urgent need for continued research on methods of elimination of LF infection among endemic and vulnerable communities. This could be achieved by an effective education program that focuses on LF transmission and prevention, via public media awareness, or by strategic advocacy on vector control. Other innovative methods of educating residents of endemic and vulnerable communities include incorporating public health professionals, audio-visual campaigns, and the running of workshops and seminars. In addition, participation in activities or exhibits that promote the adoption of policies regarding prevention and control of the disease would increase public awareness. Nevertheless, it is important that the information is presented in a concise, informative, and easy to understand form. Finally, it is recommended that an awareness campaign regarding the importance of MDA be stressed in all endemic areas of Malaysia before embarking on subsequent MDA rounds for successful implementation and control.

## Conclusion

The findings from this survey showed that there was some awareness regarding LF among people in Kemaman district of Malaysia, although knowledge of the MDA program was poor. Pre-MDA campaigns would help in improving residents’ knowledge of LF and of the purpose of MDA programs and would increase the likelihood of participation in the MDA program, thereby improving the general wellbeing of the people in the area.
